# Watching TV and Food Intake: The Role of Content

**DOI:** 10.1371/journal.pone.0100602

**Published:** 2014-07-01

**Authors:** Colin D. Chapman, Victor C. Nilsson, Hanna Å. Thune, Jonathan Cedernaes, Madeleine Le Grevès, Pleunie S. Hogenkamp, Christian Benedict, Helgi B. Schiöth

**Affiliations:** Dept. of Neuroscience, Uppsala University, Uppsala, Sweden; Paris Institute of Technology for Life, Food and Environmental Sciences, France

## Abstract

Obesity is a serious and growing health concern worldwide. Watching television (TV) represents a condition during which many habitually eat, irrespective of hunger level. However, as of yet, little is known about how the content of television programs being watched differentially impacts concurrent eating behavior. In this study, eighteen normal-weight female students participated in three counter-balanced experimental conditions, including a ‘Boring’ TV condition (art lecture), an ‘Engaging’ TV condition (Swedish TV comedy series), and a no TV control condition during which participants read (a text on insects living in Sweden). Throughout each condition participants had access to both high-calorie (M&Ms) and low-calorie (grapes) snacks. We found that, relative to the Engaging TV condition, Boring TV encouraged excessive eating (+52% g, P = 0.009). Additionally, the Engaging TV condition actually resulted in significantly less concurrent intake relative to the control ‘Text’ condition (−35% g, P = 0.05). This intake was driven almost entirely by the healthy snack, grapes; however, this interaction did not reach significance (P = 0.07). Finally, there was a significant correlation between how bored participants were across all conditions, and their concurrent food intake (beta = 0.317, P = 0.02). Intake as measured by kcals was similarly patterned but did not reach significance. These results suggest that, for women, different TV programs elicit different levels of concurrent food intake, and that the degree to which a program is engaging (or alternately, boring) is related to that intake. Additionally, they suggest that emotional content (e.g. boring vs. engaging) may be more associated than modality (e.g. TV vs. text) with concurrent intake.

## Introduction

Obesity rates have more than doubled since 1980, and in 2008 more than 1.4 billion adults were categorically overweight or obese [1]. There are a variety of lifestyle factors that have been proposed to contribute to this obesity pandemic [2]–[4]. For example, several epidemiological and laboratory studies have linked television (TV) watching to both increases in acute food intake, and subsequent weight gain and adiposity [2], [5]–[7].

While it is known that watching TV can encourage food intake, the psychological mechanisms of this effect are still poorly understood. Several studies have found that, in certain populations, television has no effect on, or even reduces concurrent food intake [8]–[10]. Importantly, each of these studies utilized unique TV programs, raising the question of whether certain types of TV may be more causative of altered eating behaviour. One possibility is that different TV programs produce different degrees of engagement or arousal, and that TV’s impact on food intake is secondary to these effects. Supporting this, recent attention has focused on the significance of emotional states, and in particular the degree of one’s boredom or arousal, in motivating eating behaviour [11]–[13]. Tying these concepts together, a recent study found that food advertisements on TV often depict negative emotional states, including boredom, on the assumption that it will promote purchasing and consumption of their product [14]. While it is known that emotional states can differentially induce food intake, there is as of yet no direct evidence linking one’s degree of engagement to TV-induced eating. Thus, we hypothesized that less engaging programs may induce greater consumption by activating a bored emotional state. Bearing this in mind, this study aimed to investigate the impact of a boring TV program vs. an engaging TV program on simultaneous food intake.

## Methods

### Ethics statement

The study was approved by the Regional Ethical Review Board in Uppsala (EPN), and the procedures followed were in accordance with the Helsinki Declaration. All participants gave written informed consent, and were reimbursed with cinema vouchers for participation in the study.

### Participants

Eighteen healthy female subjects were invited to participate (age: 22±1.3 y; body mass index: 21.1±1.1 kg/m2; all non-smokers, taking oral contraceptives). Subjects were excluded if they were currently taking any medications for physical or mental maladies (n = 2). This study population was chosen as young female participants tend to be more restrained in their eating habits, and thus more prone to experimental manipulations designed to lower restraint [15]. Additionally, this was done to avoid a ceiling effect.

### Study design and procedure

All participants engaged in three counterbalanced conditions, each lasting 30 minutes, spaced apart by one week. The three conditions included an ‘Engaging TV’ condition where participants viewed an episode of *Solsidan* (a popular Swedish comedy show, http://www.tv4.se/solsidan), a ‘Boring TV’ condition where participants viewed an art lecture on Sveriges Television (SVT, public service television, http://www.svt.se/), and a control ‘Text’ condition where participants were provided with non-engaging reading material, comprising a text on insects living in Sweden. A pilot study run with 10 women from similar demographical backgrounds established that *Solsidan* was significantly more engaging (and less boring) than both the reading and SVT conditions. Experimental sessions were scheduled not to take place during females’ respective menstruation phases.

Participants were brought in to participate at ∼7 pm, to mimic normal hours during which people watch TV, and were asked not to eat for 4 hours prior to their arrival. At the beginning of each session, participants were asked to fill out a visual analogue scale (VAS) to determine their current subjective hunger levels to confirm they had fasted. Afterwards, they participated in one of the three conditions for 30 min in total. During this period, participants had access to both high-calorie (M&Ms, 4.92 kcal/g, in total 250 grams) and low-calorie (grapes, 0.69 kcal/g, in total 300 grams) snacks, both presented in bowls. Following each session they were also administered VAS pertaining to how engaging/boring they found each condition to validate the design.

### Data analysis

Data are presented as means (±SEM). Effects of the overall intervention (Boring TV vs. Engaging TV vs. Text), the food type (grapes vs. M&Ms) and their interaction (condition*food) on the amount of food consumed were tested by means of repeated measures (within-subject) ANOVA. Additionally a linear regression was run testing for a relationship between how boring participants found the material and how much they consumed across conditions. Data were analyzed using SPSS Statistics version 17.0. Results at a p-value of ≤0.05 were considered significantly different.

## Results

### Visual analogue scale (VAS) ratings

Pre-session hunger levels did not differ significantly across conditions (P = 0.357). However, participants ratings of how engaging they found each condition did differ significantly (P<0.001). In detail, they found the Engaging TV condition significantly less boring than both the Boring TV condition (P<0.001) and the Text condition (P<0.001). However, there was no significant difference in how engaging they found the Boring TV condition and the Text condition (P = 0.424).

### Effects of condition on grams consumed

**Figure 1:**
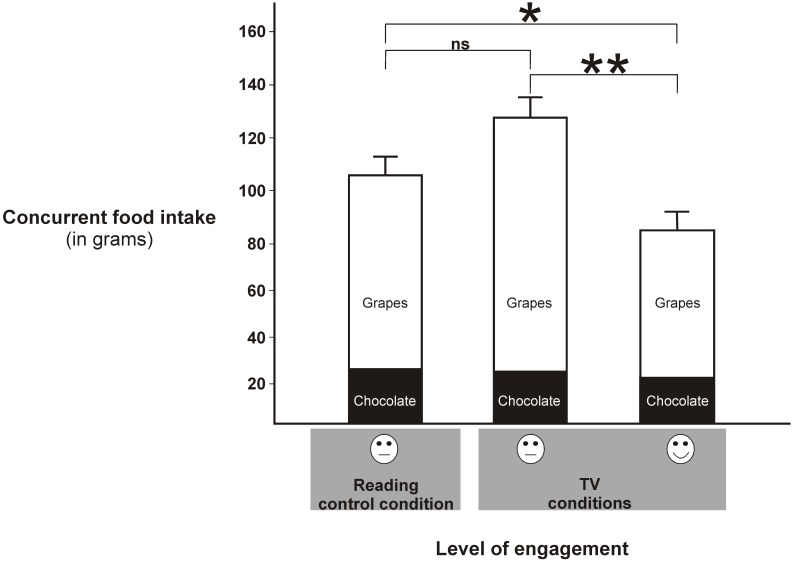
Food intake by condition. Figure detailing the total consumption (in grams) of both low calorie (grapes) and high calorie (chocolate) snacks during both Boring and Engaging (comedy) TV viewing and in the control Text condition. There was significantly more overall consumption in the Boring TV condition relative to both the Text and Engaging TV conditions. Additionally, there was significantly less gross consumption in the Engaging TV condition relative to the Text control. There was also a borderline significant interaction effect such that these changes in consumption were driven primarily by low-calorie snacks (grapes). *≤0.05, **<0.01.

In a repeated ANOVA measures analysis, there was a significant effect of condition (F(1.862) = 5.35, P = 0.01), food type (F(1) = 9.91, P = 0.006), and borderline significance in the interaction term (F(1.79) = 3.2, P = 0.06) in terms of total grams consumed. Post hoc analysis revealed that there was significantly more consumption during the Boring TV clip relative to the Engaging TV clip (125 g vs. 82 g, P = 0.009, t = −2.93, df = 17; **Figure 1**). Additionally, there was significantly more consumption in the Text condition relative to the Engaging TV condition (109.3 g vs. 81.9 g, P = 0.05, t = −2.11, df = 17; **Figure 1**). Participants also ate more in the Boring TV condition relative to the control Text condition (+15.5 grams); however, this effect did not reach significance (P = 0.252, t = 1.19, df = 17; **Figure 1**). Additionally, participants ate significantly more grams of grapes relative to M&Ms across conditions (73 g vs. 32 g, F(1) = 9.91, P = 0.006).

### Effects of condition on calories consumed

In a repeated measures ANOVA, there was no significant effect of condition found, although there was a similar pattern of results as seen in grams. There was a significant effect of food type (F(1) = 21.6, P<0.001), but no significant effect of condition, and no interaction. The effect of food type was that participants consumed significantly more calories in M&Ms than in grapes (169 kcal vs. 51 kcal, P<0.001) across conditions.

### Linear regression analysis

A linear regression analysis revealed a significant relationship between reported engagement (i.e. how engaging or boring participants found the material) and concurrent food intake across all conditions (beta = 0.317, P = 0.02). The less engaging participants found the material, the more grams of snacks they consumed.

## Discussion

The Boring TV condition induced significantly more intake of low- and high-calorie food relative to the Engaging TV condition, as measured by grams. In addition, Engaging TV actually reduced intake on both of these measures relative to the control Text condition. Boring TV also increased intake relative to the Text condition; however, these results did not reach significance. These results parallel participant consumption relative to their ratings of how boring they found each individual session irrespective of condition, as demonstrated by linear regression. Validating the design, both the control Text and the Boring TV conditions were rated as significantly more boring than the Engaging TV condition. These results demonstrate that, in our cohort of healthy young women, it is not simply TV watching that stimulates concurrent eating, but rather the content of the TV (or related media). In particular, boredom, or a lack of engagement, appears to be a significant contributor to the decision to consume under these contexts. There was also borderline significance in the interaction term, such that changes in intake were driven almost entirely by low-calorie snacks (grapes).

Early research into boredom as a possible motivator of food intake began with the premise that it would only influence obese individuals, who were prone to eat as a result of unpleasant emotional states [16]. However, it has since been established that boredom is a robust motivator of food intake in both obese and normal populations [12], [16], [17]. Additionally, these effects carry over to other disordered populations: in a study of 23 subclinical binge eaters, boredom (along with dissatisfaction with body weight and shape) was rated as the most intense proximate antecedent to binge eating episodes [18]. Following this research, this is the first study to connect boredom to consumption induced by watching TV.

While our study was motivated by the theory that watching TV induces food intake that later leads to obesity, our results do not fully resonate with that conclusion. In particular, while participants in the boring TV condition ate more, this consumption was driven almost entirely by the healthy snack (grapes) as opposed to the highly caloric M&Ms. There are several possibilities for why, in our study population, grapes were preferred when experiencing a boring stimulus. A probable explanation springs from the fact that the study population consisted of young, educated women with relatively low BMIs, which studies have demonstrated tend to be more restrained in their eating habits [15]. Thus, when induced to eat more by a stimulus, they may still retain cognizance of the type of snack they are consuming, and prefer the lower calorie option. This is particularly true in light of the fact that we theorize that it is boredom that was inducing intake in our design–boredom would theoretically have less of a distracting effect than excitement for example, leaving higher-level inhibitory processes less affected. Our study also presents some inconstancy with evidence indicating that participants tend to consume more when watching a show of their choice–presumably a pleasant, attention-grabbing show–while under our conditions they ate more in the boring condition [5]. However, the profile of our study population–young, educated women with low BMIs–could also explain why the Engaging TV condition did not elicit increases in intake. In such a population, the threshold for pleasantness to induce a reduction in restraint may be higher due to their general tendency towards restrained eating habits. These restrained eating habits could also explain why we had such a strong, reliable effect of food type, with significantly more grams of grapes being consumed in general across conditions.

Several strengths and limitations apply to the findings of this study: A within subject design was utilized, and a relatively homogenous population of young females with standard BMIs, thereby reducing variance. However, this also limits the conclusions of the study, and follow-ups in males, the elderly, children or obese populations may reveal different results. The study was conducted in a controlled laboratory environment, which again reduces variance, but in this case at the cost of external validity, as eating behavior in a food laboratory is unlikely to perfectly mimic eating behavior at home. There was no fourth condition of engaging reading material included, which may have helped to disambiguate whether the modality (TV vs. reading) as opposed to just the emotional state induced by the content (bored vs. engaged) was relevant to intake. However, this was not our primary research question, which focused on whether or not TV programs that produced different degrees of engagement would vary food intake. Finally, food intake was not measured after, only concurrent to, TV watching and reading. That said, while our findings suggest that concurrent food intake is lower when the content of the TV program being watched is an engaging comedy, it cannot be ruled out that engaging TV may increase food intake when the program is over (‘spill-over effect’) perhaps through impairment of memory for the food consumed during viewing [19].

## Conclusions

TV watching can have a significant impact on concurrent food consumption. However, it appears that the content of the TV program is elemental to this effect. While boring TV programs may encourage excessive intake, engaging TV, in the form of a comedy show, can actually reduce concurrent consumption in young adult females. This suggests that it is the degree of arousal, or the emotional state induced by TV, which modulates intake. It raises the further question of whether the modality (TV vs. reading vs. other forms of media) is relevant, so much as the content of that modality. This study further suggests that some forms of TV may in fact promote healthy eating, as there was borderline significance showing that the boring show primarily increased fruit intake. Follow up studies should investigate more precisely how the variety of emotional valences (fear, anger, calmness, etc…) modulate the role of TV and other forms of media in concurrent food intake. Additionally, studies should investigate these effects in populations other than slim, young women to test for the generality of the effect. Importantly, the reader should be aware that our study findings do not suggest that any form of TV viewing will induce long term weight loss, as TV watching is a sedentary activity.
